# Maternal carryover, winter severity, and brown bear abundance relate to elk demographics

**DOI:** 10.1371/journal.pone.0274359

**Published:** 2022-09-29

**Authors:** Sarah L. Schooler, Nathan J. Svoboda, Shannon P. Finnegan, John Crye, Kenneth F. Kellner, Jerrold L. Belant

**Affiliations:** 1 Department of Environmental Biology, State University of New York College of Environmental Science and Forestry, Syracuse, New York, United States of America; 2 Department of Fisheries and Wildlife, Michigan State University, East Lansing, Michigan, United States of America; 3 Alaska Department of Fish and Game, Wildlife Division, Kodiak, Alaska, United States of America; Texas State University, UNITED STATES

## Abstract

Ungulates are key components of ecosystems due to their effects on lower trophic levels, role as prey, and value for recreational and subsistence harvests. Understanding factors that drive ungulate population dynamics can inform protection of important habitat and successful management of populations. To ascertain correlates of ungulate population dynamics, we evaluated the effects of five non-exclusive hypotheses on ungulate abundance and recruitment: winter severity, spring nutritional limitation (spring bottleneck), summer-autumn maternal condition carryover, predation, and timber harvest. We used weather, reconstructed brown bear (*Ursus arctos*) abundance, and timber harvest data to estimate support for these hypotheses on early calf recruitment (calves per 100 adult females in July–August) and population counts of Roosevelt elk (*Cervus canadensis roosevelti*) on Afognak and Raspberry islands, Alaska, USA, 1958–2020. Increasing winter temperatures positively affected elk abundance, supporting the winter severity hypothesis, while a later first fall freeze had a positive effect on elk recruitment, supporting the maternal carry-over hypothesis. Increased brown bear abundance was negatively associated with elk recruitment, supporting the predation hypothesis. Recruitment was unaffected by spring climate conditions or timber harvest. Severe winter weather likely increased elk energy deficits, reducing elk survival and subsequent abundance in the following year. Colder and shorter falls likely reduced late-season forage, resulting in poor maternal condition which limited elk recruitment more than winter severity or late-winter nutritional bottlenecks. Our results additionally demonstrated potential negative effects of brown bears on elk recruitment. The apparent long-term decline in elk recruitment did not result in a decline of abundance, which suggests that less severe winters may increase elk survival and counteract the potential effects of predation on elk abundance.

## Introduction

Ungulates are important components of ecosystems due to their effects on lower trophic levels and role as prey, and also have economic and cultural value because of human recreational and subsistence harvests [1, 2]. Ungulate abundance and recruitment estimates are commonly used to inform population (e.g. harvest quotas) and land (e.g. timber harvest scheduling) management decisions [3, 4]. Understanding factors that drive ungulate abundance and recruitment is similarly important, as it further informs managers’ ability to protect important habitat and manage populations [5].

Numerous factors can influence ungulate population dynamics including weather, predation, forage availability and quality, and interactions among these factors [6, 7]. Determining which factors drive ungulate population dynamics is especially important with current unprecedented changes in species’ ranges and population sizes due to anthropogenic habitat alteration and climate change [8, 9], with wide-reaching implications for ecosystem and wildlife management [10]. Long-term data provide opportunities to draw stronger inference which can improve our understanding of factors that influence ungulate recruitment and population dynamics [3, 8].

Weather can affect ungulate population dynamics directly through temperature-driven physiological stress, and indirectly by altering forage availability [9]. Winter severity can reduce maternal ungulate body condition and juvenile survival through increased thermoregulatory costs from cold temperatures [11], and increased difficulty of movement or reduced forage availability due to increased snowfall [12, 13]. Spring weather also can affect ungulate population dynamics [14] because later, colder, and drier springs limit vegetation growth and availability [15]. Reduced forage quantity and quality can cause a spring nutritional “bottleneck” that may cause ungulate energetic costs to exceed reserves, reducing survival and reproductive success [16, 17]. Similarly, summers or falls with low temperatures and precipitation can reduce summer and fall forage availability, in turn reducing ungulate nutritional carryover, which reduces pregnancy rates, winter survival, body condition, and ultimately abundance and recruitment [18, 19]. Predators also affect ungulate survival [20, 21], which influences long-term abundance and recruitment [3, 22]. Anthropogenic habitat alterations such as timber harvest influence ungulate habitat suitability, and therefore abundance and recruitment [23, 24]. Recently harvested timber stands can increase habitat suitability by increasing forage availability [4, 25] and young stands can increase habitat suitability by increasing hiding cover [4], however timber harvest also reduces mature forest area, which may provide shelter from predation and severe winter weather [26, 27].

To quantify factors that limit ungulate populations, we tested five non-exclusive hypotheses to assess their effects on Roosevelt elk (*Cervus canadensis roosevelti)* early calf recruitment (defined as calves per 100 adult females in July–August; henceforth recruitment) and abundance (Table 1). The winter severity hypothesis predicts that cold temperatures or high snowfall cause decreased abundance and recruitment [12, 21]. The spring bottleneck hypothesis predicts that a prolonged winter or a spring that is cool or dry leads to decreased abundance and recruitment [17, 28]. The maternal carryover hypothesis predicts that a short, cool, or dry summer; a cool or dry autumn; or an early first freeze causes decreased abundance and recruitment the following summer [18]. The predation hypothesis predicts that increasing abundance of brown bears will decrease elk recruitment, as brown bears may predate elk calves [3, 19, 21]. Finally, the timber harvest hypothesis predicts that timber harvest will alter elk habitat suitability, and therefore recruitment [4, 29], in that an increase in area of recently harvested timber stands will increase forage availability and therefore positively influence recruitment, or alternatively, an increase in area of recently harvested timber stands will reduce availability of mature forest and therefore negatively affect recruitment [4].

**Table 1:** Hypotheses, predictions, and covariates used to estimate factors influencing elk abundance and recruitment.

Hypothesis	Prediction	Covariates
1) Winter severity:		
a) Temperature	As winter temperatures decrease, abundance and recruitment decrease	Mean winter temperature, mean monthly minimum winter temperature (November–March)
b) Snow	As snow depth or total snowfall increases, abundance and recruitment decrease	Mean snow depth, total snowfall (November–March)
2) Spring bottleneck:		
a) Spring quality	As spring temperature and/or precipitation increases, abundance and recruitment increase	Mean spring temperature, total spring precipitation (April–May)
b) Spring timing	As the last spring freeze is later, abundance and recruitment decrease	Last spring freeze
c)Winter duration	As winter duration increases, abundance and recruitment decrease	Winter duration (# days between first fall freeze and final spring freeze)
3) Maternal carry-over:		
a) Summer quality	As growing degree days, summer temperatures and/or summer precipitation increase, abundance and recruitment increase in the following year	Mean summer temperature, total summer precipitation, growing degree days (June–August; t-1)
b) Summer Productivity	As SPEI decreases, abundance and recruitment increase in the following year	6-month SPEI calculated in July (forest SPEI), 3-month SPEI calculated in September (grassland SPEI) (t-1)
c) Fall quality	As fall temperatures and/or precipitation increase, abundance and recruitment increase in the following year	Mean fall temperature, total fall precipitation (September–October; t-1)
d) Fall timing	As the first fall freeze is later, abundance and recruitment increase in the following year	First fall freeze (t-1)
4) Predation:		
	As abundance of brown bears increases, recruitment decreases	Reconstructed brown bear abundance
5) Timber Harvest:		
a) Timber harvest increases habitat suitability	As area of timber harvest or young timber stands increases, recruitment increases	Square kilometers of timber stands age <1, 1–5, 6–30, and >30 years since harvest, square kilometers of all harvested area
b) Timber harvest decreases habitat suitability	As area of timber harvest or young timber stands increases, recruitment decreases	Square kilometers of timber stands age <1, 1–5, 6–30, and <30 years since harvest, square kilometers of all harvested area

Hypotheses and predictions were tested on elk early calf recruitment (calves per 100 adult females in July–August) and population counts of elk on Afognak and Raspberry islands, Alaska, USA. Predictors for the year prior to the composition and population counts are represented as t-1. SPEI is the standardized precipitation evapotranspiration index (see methods for details).

## Materials and methods

### Study area

**Fig 1:**
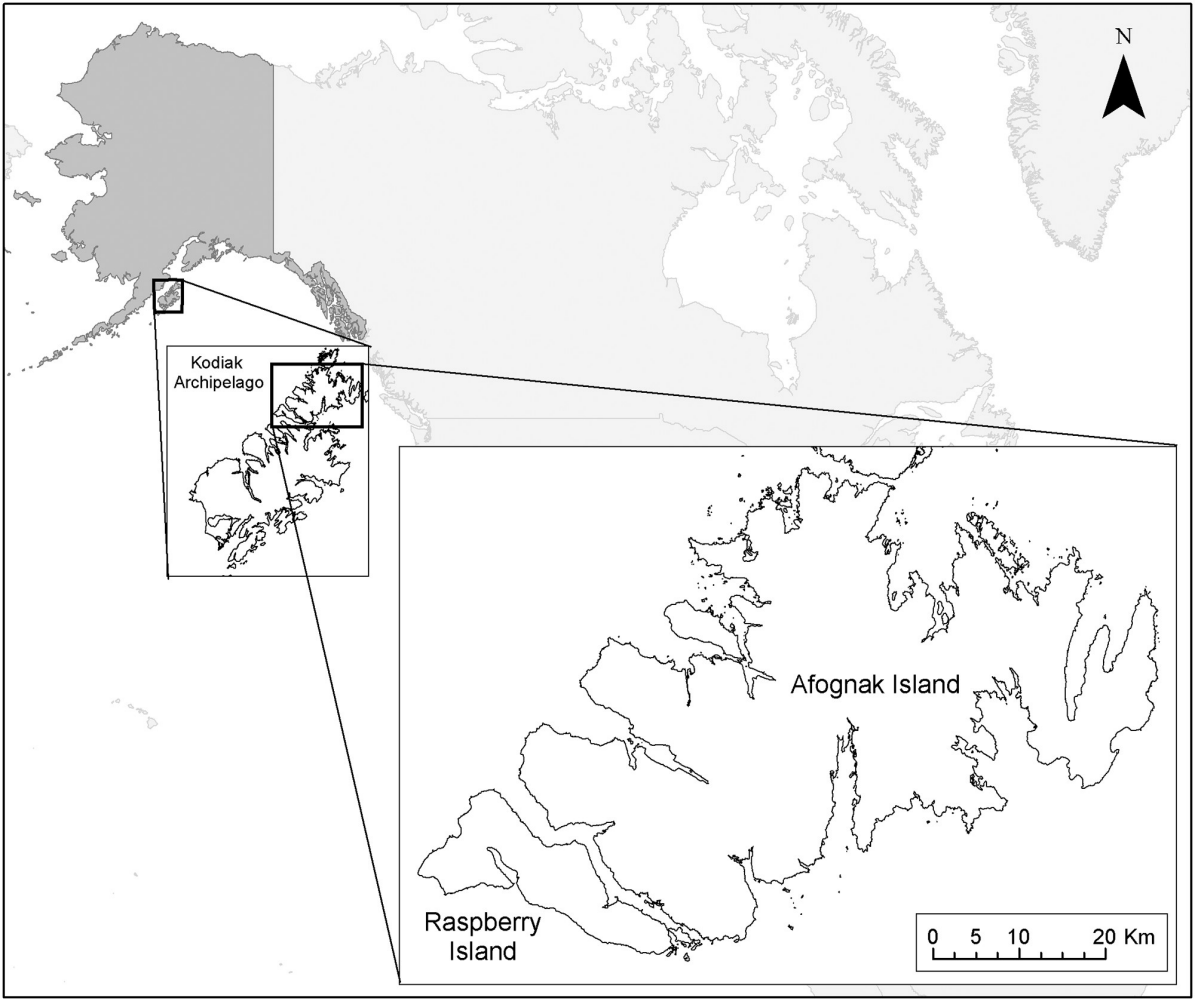
Afognak and Raspberry islands, Alaska, USA. World and state map outlines from NASA open data portal. Afognak and Raspberry islands outline from the Kodiak Island Borough maps and data center.

Afognak (1,809 km^2^; 58.3279° N, 152.6415° W) and Raspberry (197 km^2^; 58.0708° N, 153.1876° W) islands are in the Kodiak Archipelago, Alaska, USA (Fig 1). The islands are 5 km north of Kodiak Island and separated by a 1.5-km wide strait. Afognak and Raspberry islands are primarily owned by Native corporations (64%), followed by state (27%) and federal (9%) ownership. Both islands contain gradual sloping mountains ranging from 300 to 800 m in elevation. The archipelago has a subpolar oceanic climate with average annual high and low temperatures of 8.0ºC and 2.1ºC, respectively [30]. Average annual rainfall and snowfall are 174 cm and 172 cm, respectively [30].

**Fig 2:**
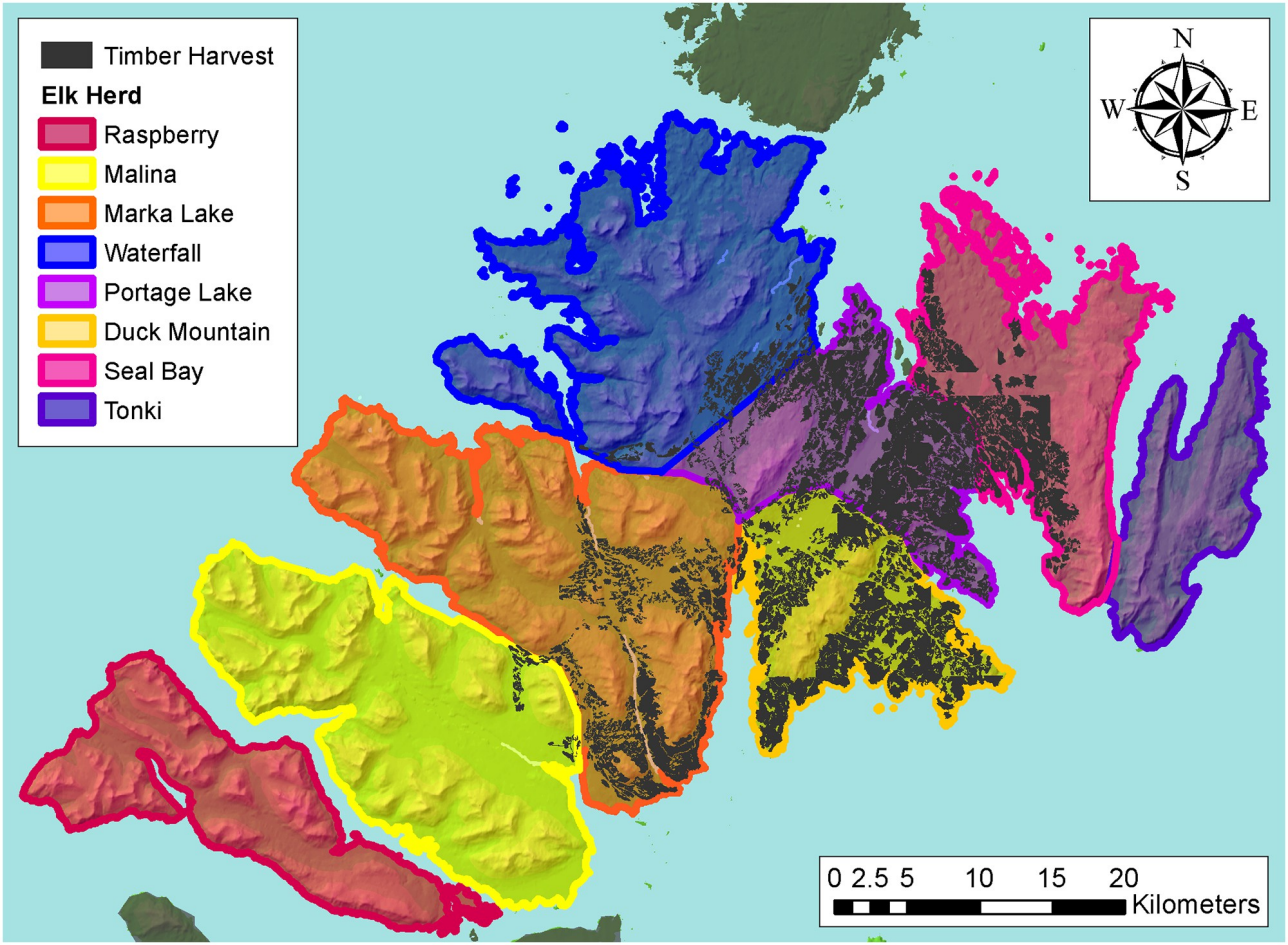
Area of timber harvest and locations of elk herds, Afognak and Raspberry islands, Alaska, USA. Background terrain map from Alaska Department of Fish and Game Wildlife Division Staff.

The eastern portion of Afognak Island is dominated by Sitka spruce (*Picea sitchensis*) which occurs up to 365 m in elevation with an understory containing blueberry (*Vaccinium ovalifolium*), devil’s club (*Oplopanax horridus*), salmonberry (*Rubus spectabilis*), and elderberry (*Sambucus racemosa*) [31, 32]. Other portions of the island are dominated by alder (*Alnus fruiticosa*) and willow (*Salix* spp.) interspersed with open herbaceous areas containing forbs such as bluejoint (*Calamagrostis canadensis*) and fireweed (*Epilobium angustifolium*) [31, 32]. Raspberry Island has primarily herbaceous meadows with some alder and willow and limited spruce [31]. Small portions of Afognak Island were logged intermittently during 1930–1965 [33], with extensive commercial logging on south-central Afognak Island since 1977 (Fig 2). No commercial logging has occurred on Raspberry Island since the 1930s.

In 1929, eight Roosevelt elk were introduced to Afognak Island by the Alaska Game Commission to establish a harvestable population [32]. The population increased to over 1000 animals by 1965 and has since varied annually from 700 to 1200 individuals [34, 35]. Elk on Afognak Island are divided into seven herds that intermix, especially during winter, and an eighth herd occurs on Raspberry Island which has little interchange with other herds [34, 36].

Black-tailed deer (*Odocoileus hemionus*) are the only other ungulate on Afognak and Raspberry islands. There are an estimated 70,000–75,000 black-tailed deer on the Kodiak Archipelago, which includes an unknown number on Afognak and Raspberry islands [37]. Though black-tailed deer and elk diets overlap, elk can outcompete deer because of their larger size and ability to consume coarser plant material [38, 39]. Brown bears (*Ursus arctos*) are the only non-human predator of elk on the islands [34], and when predating ungulates, typically consume elk calves [40]. Brown bears are considered common on Afognak and Raspberry islands but there are no recent estimates of abundance [41]. Brown bears, elk, and black-tailed deer can be legally harvested with permits issued by the Alaska Department of Fish and Game (ADFG) [42]. Number of harvest permits issued is determined using population size estimates and harvest rates in the previous year [35, 43].

### Elk data

To measure elk early calf recruitment and abundance, we used aerial surveys conducted sporadically during 1958–1964 and annually thereafter [35]. Elk counts were conducted by ADFG biologists twice per year, first during July–August, emphasizing on herd composition to measure early calf recruitment (calves per 100 adult females; age ratios), and then during September, to estimate population size [35]. Surveys were conducted with no set flight plan at various altitudes to maximize elk sightability and identification, and counts were conducted independently by the pilot and biologist until a consensus was reached [35]. For composition counts, adult females, males and calves were identified by size, antler presence, and coloration [44]. During 1992–2020, locations of elk herds for surveys were obtained with very high frequency radio- or Global Positioning System (GPS) collars deployed as part of a parallel study [35].

While extensive canopy cover limited efficacy of herd abundance counts on Afognak Island, composition counts only require a portion of the herd to be observed, with the assumption that the portion observed is representative of the herd, and therefore can be conducted in areas where population counts are not feasible [45]. Because Raspberry Island has little canopy cover, we were able to incorporate elk abundance for the Raspberry herd only. We accounted for elk harvest on Raspberry Island when modeling elk abundance. Elk harvest occurs during fall after population counts (e.g. 25 September–30 November in 2021) and harvest information has been collected by ADFG since 1949 [42].

### Climate data

We used weather data from Kodiak Airport [30, 46], 35 km south of Afognak Island, to derive predictor variables to test for support of the winter severity, spring bottleneck, and maternal carryover hypotheses (Table 1). For the winter severity hypothesis, we tested mean temperature, mean monthly minimum temperature, total snowfall, and mean daily snow depth during winter (1 November–31 March). For the spring bottleneck hypothesis, we tested mean spring (1 April–31 May) temperature, total spring precipitation, date of last spring freeze, and the number of days between the first fall freeze and last spring freeze (i.e., winter duration). For the maternal carryover hypothesis, all predictors were from the summer and autumn in the year before the respective population count and composition surveys. We tested mean summer (1 June–31 August) temperature, total summer precipitation, growing degree days calculated at base 5°C [47], the standardized precipitation evapotranspiration index (SPEI) [48], mean fall (1 September–31 October) temperature, total fall precipitation, and date of first fall freeze.

In boreal regions, SPEI is negatively correlated with summer productivity [49]. We used R package SPEI to calculate potential evapotranspiration using the “thornthwaite” function [50] and “spei” to calculate SPEI with a calibration period during January 1950–May 2021 [48]. We used two SPEI calculations: a 6-month period (January–July) which best predicts forest productivity (forest SPEI), and a 3-month period (July–September) which best predicts grassland productivity (grassland SPEI) [51].

### Brown bear data

Brown bear harvests on the Kodiak Archipelago occur during fall and spring (25 October–30 November and 1 April–15 May) [42]. Hunters are limited to one bear per permit and harvest of females with dependent young is prohibited. All harvested bears are inspected by ADFG personnel who record sex and location of kill for each bear and extract a vestigial premolar tooth for cementum aging [52]. Additionally, ADFG inspects and extracts teeth for all other known brown bear mortalities (e.g. illegal harvest, defense of life and property, vehicle or natural mortalities).

We used Downing population reconstruction to estimate brown bear abundance on Afognak and Raspberry islands using total harvest by year and harvest-by-age data [53, 54]. Downing population reconstruction assumes that the primary source of mortality is harvest, but we augmented our data by including all known mortalities [53]. Downing reconstruction also implies that cohort harvest mortality relative to total mortality is constant among years, that mortality rates for the oldest two reconstructed age classes are equal, and that the aged sample is unbiased, but Downing’s method is robust to violations of many of these assumptions [54]. Additionally, because this brown bear population has a low rate of natural mortality with little interannual variability [41], a high harvest reporting rate [55] and a high and unbiased rate of aging of harvested bears (average number of teeth aged relative to bears harvested = 0.92), we believe that the Downing population reconstruction provided reasonable estimates of brown bear abundance trends.

Cementum aging records were available from 1967 to 2017 (S1 Dataset). Though brown bears do not achieve full body size until 8–14 years old [56], Downing population reconstruction is robust to collapsing age classes [54]. Downing population estimates calculated from these data were similar using 5, 7, and 12 age classes. We therefore used five age classes to increase the number of years available for analyses. As Downing population reconstruction provides inaccurate abundance estimates for one less than the number of age classes estimated in most recent years [53], we excluded the four most recent years of abundance estimates (2014–2017) from our analysis. We did not analyze sexes separately because a preliminary Downing reconstruction using all known mortalities of bears in the Kodiak Archipelago revealed no difference in abundance estimates when sexes were analyzed separately (average yearly proportional difference in summed sex-separated estimate compared to sexes combined = 0.022).

### Timber harvest data

We compiled 45 years (1976–2020) of timber harvest data from Afognak Native Corporation, Koniag Native Corporation, Koncor, Natives of Kodiak Native Corporation, and Ouzinkie Native Corporation. We verified, corrected, and added to these data using Google Earth and Landsat satellite imagery [57, 58]. We characterized forest stand by age based on their habitat value for elk including clear-cut (<1 year), early regeneration (1–5 years), late regeneration (6–30 years), and mature forest (>30 years) [59–61], then calculated the total area harvested and area of each age class in each year (Table 1).

### Analysis

Using location data from GPS-collared elk [39], we separated recruitment data based on elk herds which occurred in areas with timber harvest (Marka Lake, Waterfall, Portage Lake, Duck Mountain, Seal Bay; “timber harvest dataset”) and areas without timber harvest (Raspberry, Malina; “non-timber harvest dataset”; Fig 2) by summing calf and cow counts from the respective herds then calculating the number of calves per 100 adult females (hereafter age ratio). We also used a similarly calculated combined dataset of all herds except Tonki as dense forest cover precluded accurate composition counts (“island-wide dataset”). Before summation of the recruitment datasets, we removed herd counts for years in which the number of females was less than the number of calves, because this indicated an error in data recording or incomplete count of the grouping; the number of calves counted was zero, because these indicated that the survey was conducted too late in the year to distinguish cows from calves; and years in which cows and bulls were not counted separately (S1 Table). We excluded the recruitment count in 2006 from the timber harvest dataset because the total number of elk counted across all herds was five. Given these exclusions, we did not analyze recruitment data for 1998, 2006, or 2007 (S2 Dataset). We standardized all predictor variables by mean and standard deviation to compare effect sizes [62].

We fit linear models to each elk dataset (timber recruitment, non-timber recruitment, island-wide recruitment, and Raspberry herd abundance) with year as the predictor, and used p-values (α < 0.05) to determine long-term trends [62]. To determine long-term trends in estimated brown bear abundance, we also fit linear models to the brown bear population reconstruction and natural mortality rates, also using p-values (α < 0.05).

The winter severity, spring bottleneck, maternal carryover, and timber harvest hypotheses were represented by several intercorrelated predictor variables that had similar biological meanings (Table 1; S1 File). To determine which measured predictor variable best represented the corresponding hypothesis, we first fit univariate regression models for each predictor to determine which had the strongest relationships with the response variables (i.e. recruitment and abundance). Within each hypothesis, we then selected the predictor with the greatest R^2^ value to represent the respective hypothesis in the second stage of the analyses [62, 63]. We examined the correlation between selected predictors using Pearson’s product-moment correlation coefficient (*r*). For those highly correlated (|*r*| > 0.7), we replaced the correlated predictor with an uncorrelated predictor with the next greatest R^2^ value that allowed us to test the maximum number of hypotheses [62, 63].

To test our hypotheses on recruitment, we used the selected predictors for each hypothesis to fit Bayesian general linear models to the timber harvest recruitment, non-timber harvest recruitment, and island-wide recruitment datasets (S1 Appendix). Area of timber harvest was included only in the timber harvest and island-wide recruitment models.

To test our hypotheses on changes in Raspberry herd abundance, we used the selected predictors for each hypothesis to fit a Bayesian Gompertz state-space model to the time series of elk count data, which accounts for potential density dependence and has been used previously to assess drivers of ungulate population dynamics [7, 12, 64]. We also explicitly incorporated known harvest size in each year [64]. We did not include reconstructed brown bear abundance as a covariate for this model because we were unable to accurately estimate brown bear abundance on Raspberry Island. Population count data were imputed using linear interpolation for 1959, 1960, 1963, and 2007 (S2 Dataset). A more detailed description of the model can be found in S1 Appendix. Because the coefficient for density dependence in the Gompertz model with external predictors is only partially identifiable even within a state-space context [65, 66], we also tested for density dependent effects within the Raspberry herd using the Dennis-Taper parametric bootstrap likelihood ratio t-test, and examined a plot of the natural log of the per capita growth rate versus abundance [66, 67].

We used Bayesian methods for all models because they allow for clear interpretation of results and have better inference with smaller sample sizes [68]. We fit models using Gibbs sampling through a wrapper for the R package rjags, jagsUI (annotated jagsUI code provided in S2 File) [69, 70]. We used uninformative normally distributed (for parameters) or uniformly distributed (for error) priors with three chains, a thin rate of 5, and 50,000 iterations with a burn-in of 10,000. We examined traceplots, residual plots, scale reduction factors ($\hat{R}$), and Bayesian p-values to assess model convergence and goodness of fit, respectively, and used 95% credible intervals to determine influence of predictors [68].

## Results

Annually, an average of 20±8.9 (standard deviation) known brown bear mortalities occurred on Afognak and Raspberry islands during 1967–2017 (S1 Dataset). Average annual reconstructed brown bear abundance on Afognak and Raspberry islands was 163±76.2 bears, ranging from 83 to 331 individuals (S1A Fig). Overall annual brown bear abundance increased by 4.7 individuals (R^2^ = 0.729, p < 0.001, t = 11.00). Known brown bear mortalities remained steady during 1967–1993 after which they increased until 2008 (2002–2008 estimate = 1.07, R^2^ = 0.497, p = 0.002, t = 3.846).

We used 39 years of age ratio data during 1967–2013 from the Waterfall, Duck Mountain, Marka Lake, Portage Lake, and Seal Bay herds to assess effects of timber harvest on elk recruitment. The elk age ratio ranged from 16 to 48 calves per 100 adult females, with a mean age ratio of 33±7.9 calves per 100 adult females and demonstrated no long-term trend (estimate = -0.15, R^2^ = 0.043, p = 0.104, t = -1.664; S2 Fig). For the timber harvest hypothesis, the two predictors with the greatest R^2^ values were correlated with brown bear abundance (timber harvest aged 6–30, *r* = 0.955; total area harvested, *r* = 0.927; Table 2; S1 File), therefore we used area of timber harvest aged 1–5. None of the other selected predictors (i.e. those with the greatest R^2^ values for each hypothesis) were highly correlated (all |*r*| < 0.7) and therefore were used in the final model. The final model included mean winter temperature representing the winter severity hypothesis, winter duration representing the spring bottleneck hypothesis, day of first fall freeze representing the maternal carryover hypothesis, brown bear abundance representing the predation hypothesis, and area of timber harvest aged 1–5 for the timber harvest hypothesis. The final model successfully converged (all $\hat{R}$ < 1.1) and fit well (Bayesian p-value = 0.538), but there was no support for any of the predictor variables, as all 95% credible intervals overlapped zero (Table 3).

**Table 2:** Standardized regression coefficients (R^2^) from univariate models predicting elk recruitment and abundance.

Hyp.	Predictor	Timber	Non-timber	Island-wide	Raspberry
Winter Severity	Mean min. winter temperature (°C)	0.002	**0.035**	**0.005**	0.199
	Mean winter temperature (°C)	**0.006**	0.014	<0.001	**0.212**
	Snowfall (mm)	0.006	0.013	<0.001	0.098
	Snow depth (mm)	0.001	0.007	0.001	0.109
Spring Bottleneck	Mean spring temperature (°C)	0.016	**0.062**	0.008	**0.106**
	Spring precipitation (mm)	0.026	0.002	0.009	0.005
	Day of final spring freeze (°C)	0.032	0.002	0.020	0.016
	Winter season length (days)	**0.121**	0.021	**0.100**	0.030
Maternal Carry-over	Mean summer temperature (°C)	0.041	0.016	0.001	0.128
	Summer precipitation (mm)	0.074	0.008	0.033	0.010
	Growing degree days	0.022	0.001	0.003	0.181
	SPEI forest	0.129	<0.001	0.022	0.017
	SPEI shrub	0.018	<0.001	0.002	0.061
	Mean fall temperature (°C)	0.018	0.025	0.029	**0.186**
	Fall precipitation (mm)	0.074	0.008	0.056	0.017
	Day of first fall freeze	**0.180**	**0.059**	**0.175**	0.016
Timber Harvest	Area timber harvest age < 1 (km^2^)	0.000		0.025	
	Area timber harvest age 1–5 (km^2^)	**0.037**		**0.064**	
	Area timber harvest age 6–30 (km^2^)	0.052		0.222	
	Area timber harvest age > 30 (km^2^)	0.008		0.048	
	Total area timber harvest (km^2^)	0.050		0.193	
	Brown bear abundance	**0.033**	**0.224**	**0.203**	

Standardized regression coefficient (R^2^) values for elk univariate linear models for all predictors for each hypothesis (Hyp.) and model (timber recruitment [timber], non-timber recruitment [non-timber], island-wide recruitment [island-wide], and Raspberry herd abundance [Raspberry]) with selected predictors for each model in bold, Afognak and Raspberry islands, Alaska, USA, 1958–2020.

**Table 3:** Results from timber, non-timber, and island-wide elk recruitment and abundance models.

Model	Predictor	Mean	SD	2.50%	97.50%
Timber recruitment	Intercept	32.684	1.270	30.191	35.154
	Standard deviation	7.845	1.026	6.147	10.176
	Mean winter temperature (°C)	0.293	1.365	-2.339	2.934
	Winter duration (days)	-1.014	1.685	-4.321	2.319
	Day of first fall freeze	2.719	1.722	-0.631	6.166
	Brown bear abundance	0.506	1.720	-2.876	3.896
	Area of timber harvest aged 1–5 (km^2^)	-0.638	1.601	-3.807	2.469
Non-timber recruitment	Intercept	33.266	1.257	30.788	35.751
	Standard deviation	8.272	0.974	6.633	10.436
	Mean minimum winter temperature (°C)	-1.893	1.405	-4.675	0.854
	Mean spring temperature (°C)	0.035	1.696	-3.229	3.397
	Day of first fall freeze	1.755	1.630	-1.460	4.968
	**Brown bear abundance**	**-4.375**	**1.341**	**-6.913**	**-1.760**
Island-wide recruitment	Intercept	32.667	0.981	30.769	34.612
	Standard deviation	6.441	0.777	5.132	8.177
	Mean minimum winter temperature (°C)	-1.128	1.046	-3.165	0.980
	Winter duration (days)	-0.767	1.332	-3.334	1.823
	**Day of first fall freeze**	**2.801**	**1.347**	**0.205**	**5.466**
	**Brown bear abundance**	**-3.418**	**1.228**	**-5.889**	**-1.074**
	Area of timber harvest aged 1–5 (km^2^)	1.765	1.416	-0.983	4.581
Raspberry abundance	Intercept	1.453	0.372	0.757	2.214
	Observation error	0.205	0.053	0.102	0.307
	Process error	0.229	0.060	0.116	0.349
	**Mean winter temperature (°C)**	**0.136**	**0.053**	**0.031**	**0.240**
	Mean spring temperature (°C)	0.018	0.046	-0.071	0.110
	Mean fall temperature (°C)	-0.023	0.056	-0.134	0.087
	**Density dependence**	**-0.267**	**0.083**	**-0.442**	**-0.120**

Model results from the timber recruitment (n = 39), non-timber recruitment (n = 44), island-wide recruitment (n = 44), and Raspberry herd abundance (n = 63) models showing estimated predictors, scaled mean model coefficients, standard deviations (SD), and credible intervals (2.5%, 97.5%), with significant predictors in bold, Afognak and Raspberry islands, Alaska, USA, 1958–2020.

**Fig 3:**
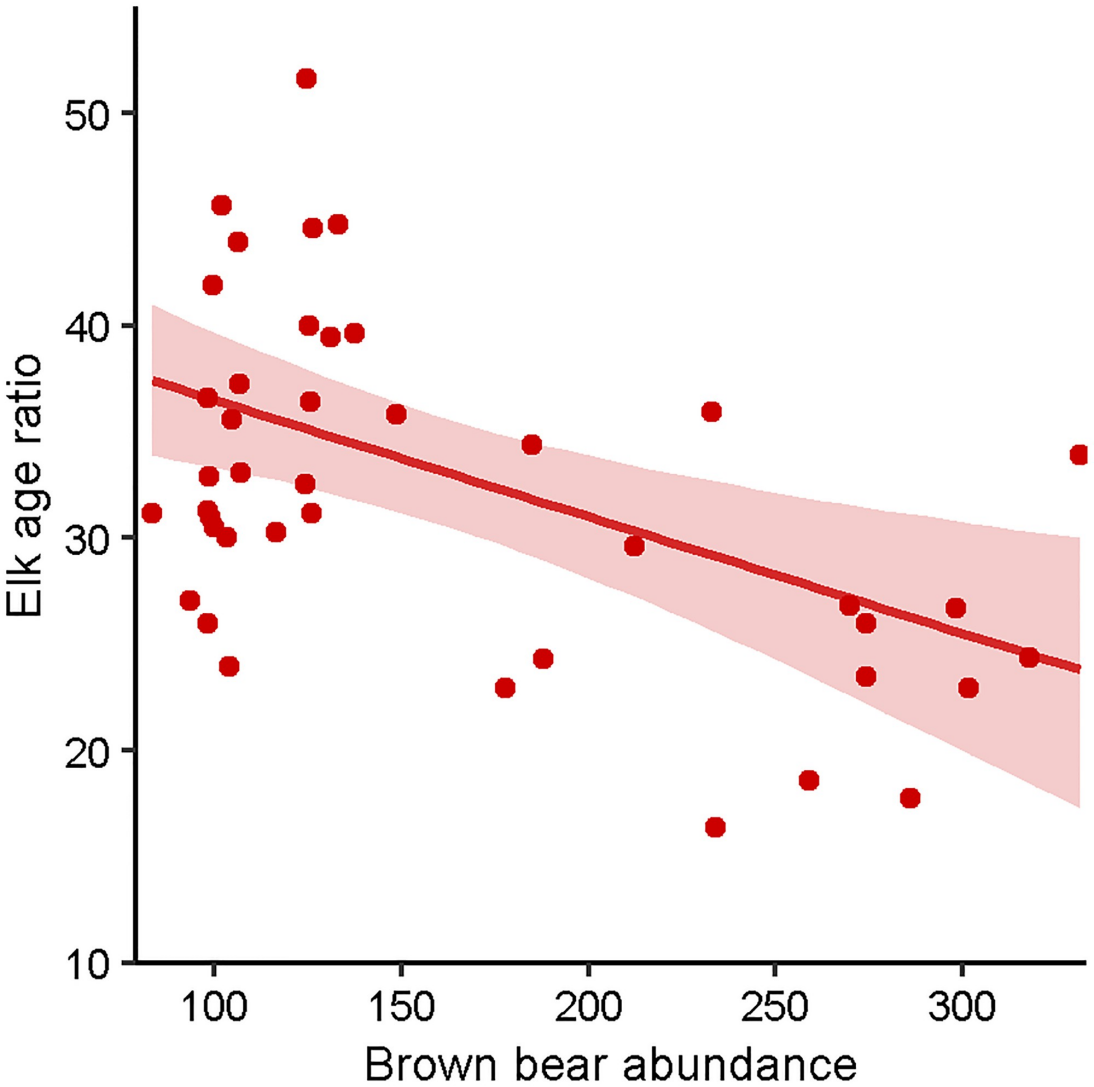
Elk age ratios predicted by brown bear abundance by the non-timber harvest recruitment model. Model-predicted response of semi-annual elk age ratios (calves per 100 adult females) in non-timber harvest area as a function of brown bear abundance with 95% credible interval (shading), Afognak and Raspberry islands, Alaska, USA, 1967–2013.

We used 44 years of age ratio data during 1967–2013 from the Malina and Raspberry herds to assess drivers of elk recruitment in areas without timber harvest. The elk age ratio ranged from 16 to 57 calves per 100 adult females, with a mean ratio of 33±9.4 calves per 100 adult females (S2 Fig). The ratio decreased by 0.32 calves per 100 adult females annually (R^2^ = 0.185, p = 0.001, t = -3.316). None of the selected predictors (i.e. those with the greatest R^2^ values for each hypothesis) were correlated (all |*r*| < 0.7; S1 File) and therefore all were used in the final model. The final model included mean monthly minimum winter temperature representing the winter severity hypothesis, mean spring temperature representing the spring bottleneck hypothesis, day of first fall freeze representing the maternal carry-over hypothesis, and brown bear abundance representing the predation hypothesis (Table 2). The final model successfully converged (all $\hat{R}$ < 1.1) and fit well (Bayesian p-value = 0.528). Brown bear abundance was the only influential predictor, as all other predictors had 95% credible intervals overlapping zero (Table 3). Predicted elk age ratio decreased by 13.6 calves per 100 adult females across observed brown bear abundance (83–331 individuals; Fig 3).

**Fig 4:**
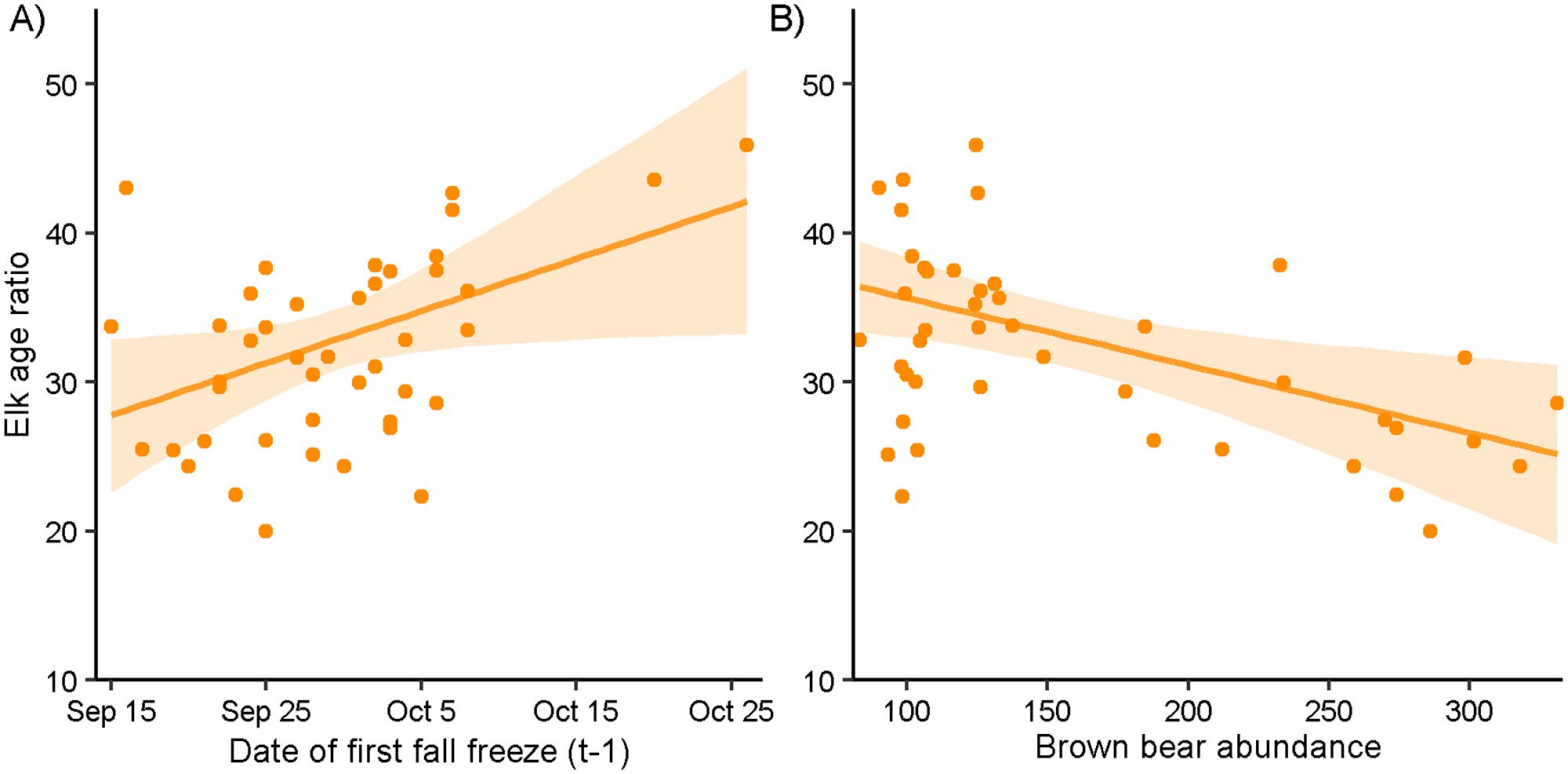
Island-wide elk age ratios predicted by date of first fall freeze and brown bear abundance. Model-predicted response of semi-annual island-wide elk age ratios (calves per 100 adult females) as a function of (A) date of first fall freeze in the previous year and (B) brown bear abundance with 95% credible intervals (shading), Afognak and Raspberry islands, Alaska, USA, 1967–2013.

We used 44 years of age ratio data during 1967–2013 from all elk herds in the island-wide recruitment model. The elk age ratio ranged from 20 to 57 calves per 100 adult females, with a mean ratio of 33±7.2 calves per 100 adult females. The ratio decreased by 0.22 calves per 100 adult females annually (R^2^ = 0.165, p = 0.003, t = -3.116; S2 Fig). The two predictors with the greatest R^2^ values for the timber harvest hypothesis were correlated with brown bear abundance (timber harvest aged 6–30 years old *r* = 0.959, total area harvested *r* = 0.929; Table 2; S1 File), so we used area of timber aged 1–5 for the timber harvest hypothesis. None of the other selected predictors (i.e. those with the greatest R^2^ values for each hypothesis) were correlated (all |*r*| < 0.7) and thus were used in the final model. The final island-wide recruitment model included mean monthly minimum winter temperature representing the winter severity hypothesis, winter duration representing the spring bottleneck hypothesis, day of first fall freeze representing the maternal carry-over hypothesis, brown bear abundance representing the predation hypothesis, and area of timber harvest aged 1–5 for the timber harvest hypothesis. The final model successfully converged (all $\hat{R}$ < 1.1) and fit well (Bayesian p-value = 0.532). Brown bear abundance and date of first fall freeze were the only influential predictors, as other 95% credible intervals overlapped zero (Table 3). The predicted elk age ratio increased as first fall freeze occurred later in the year, increasing by 14.3 calves per 100 adult females across the range of observed values (17 September–27 October; Fig 4A). As brown bear abundance increased, the predicted elk age ratio decreased by 11.2 calves per 100 adult females across the range of observed values (83–331 individuals; Fig 4B). The negative effects of increasing brown bear abundance on elk recruitment (β = -3.42, 95% credible interval = -5.89, -1.07) were greater than the positive effects of a later fall freeze (β = 2.80, 95% credible interval = 0.21, 5.47).

**Fig 5:**
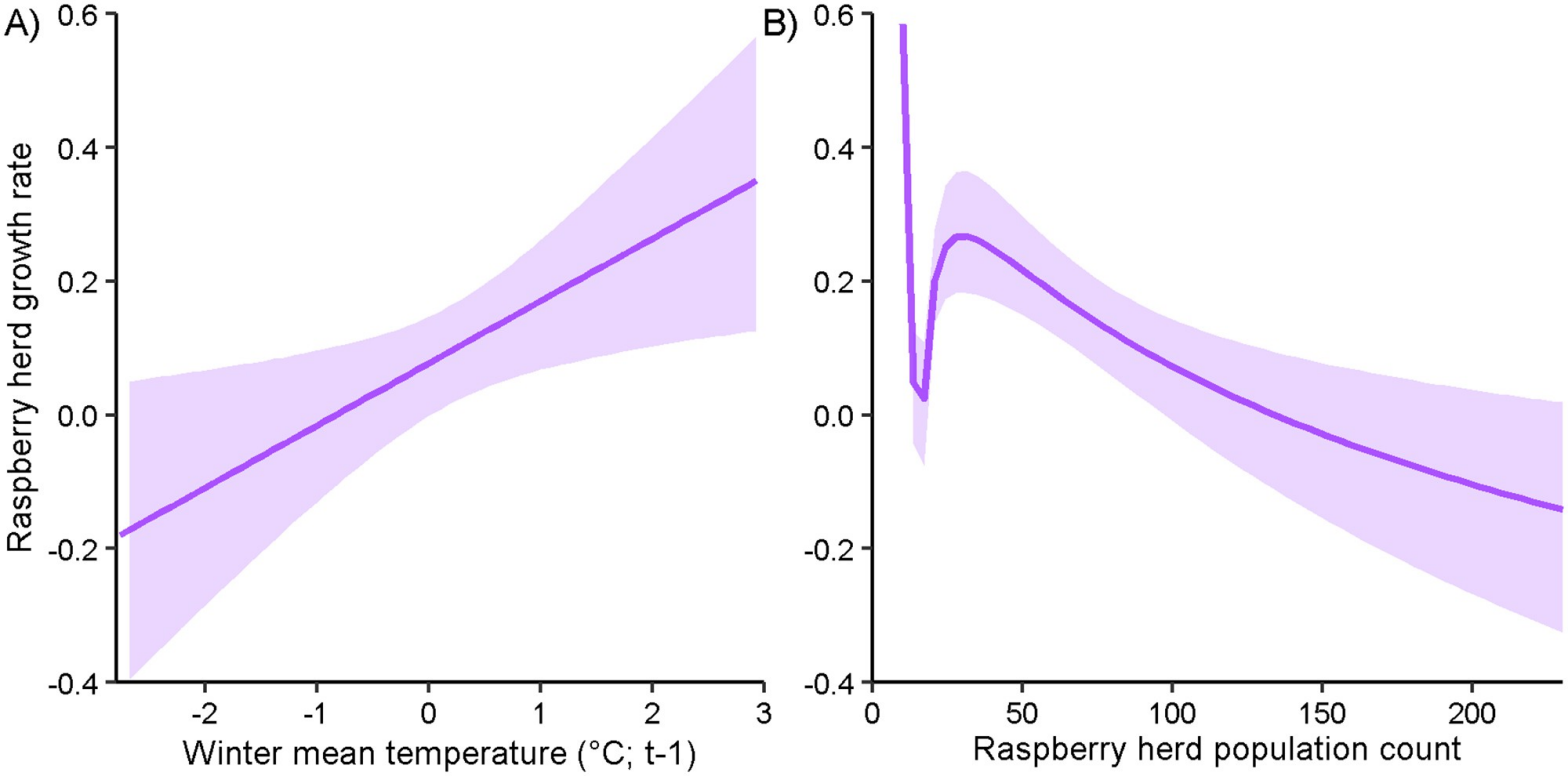
Elk Raspberry herd growth rate predicted by mean winter temperature and population count. Model-predicted response of semi-annual Raspberry herd abundance as a function of (A) mean winter temperature in the previous year and (B) elk population count, with 95% credible intervals (shading), Raspberry Island, Alaska, USA, 1958–2020.

We used 63 years of aerial count data during 1958–2020 for the Raspberry herd state-space abundance model. Raspberry herd population counts ranged from 10 to 230 elk with a mean of 116±61.7 elk (mean density = 0.59±0.31 elk/km^2^), and abundance appeared cyclical with no evidence of long-term trend (estimate = 0.723, R^2^ = 0.026, p = 0.117, t = 1.594; S3 Fig). None of the selected predictors (i.e. those with the greatest R^2^ values for each hypothesis) were correlated (all |*r*| < 0.7; S1 File) and therefore all were used in the final model. The final model for growth rate included mean winter temperature representing the winter severity hypothesis, mean spring temperature representing the spring bottleneck hypothesis, mean fall temperature representing the maternal carryover hypothesis, and a parameter for density dependence (Table 2). The final model successfully converged (all $\hat{R}$ < 0.01) and fit well (Bayesian p-value = 0.528). Mean winter temperature and the parameter associated with density dependence were both influential predictors; all others had 95% credible intervals overlapping zero (Table 3). As mean winter temperature increased, predicted growth rate increased by 0.53 over the range of observed values (-2.80–2.93°C; Fig 5A). Though the credible interval of the density dependence parameter did not overlap zero (Fig 5B), the Dennis-Taper parametric bootstrap likelihood ratio t-test was insignificant, indicating lack of density dependence (p = 0.336; S4 Fig).

## Discussion

Weather explained variation in elk abundance and recruitment. The winter severity hypothesis was supported by the Raspberry herd abundance model, while the maternal carryover hypothesis was supported by the island-wide recruitment model. We found no support for the spring bottleneck hypothesis, but we found the predation hypothesis received the greatest overall support in explaining recruitment island-wide and in areas without timber harvest. We found no support for the timber harvest hypothesis.

We found that elk abundance was affected by winter temperature, supporting the winter severity hypothesis. Increased winter severity, in the form of increased snowfall [21], snow depth [13], and decreased temperatures [71] can have a negative effect on elk survival across a broad range of climatic conditions, which in turn leads to decreased abundance after harsh winters [72]. Though severe winters and later, colder springs can be detrimental to ungulate recruitment [14, 73, 74], our findings on early calf recruitment did not support the winter severity or spring bottleneck hypotheses, indicating that winter severity has an impact on overwinter survival, rather than successful parturition and survival of calves to two months.. Winter may have less impact on early calf parturition for northern ungulates in coastal areas with milder winters and warmer summers than typical mainland subpolar and continental climates [6, 75]. For example, caribou early calf recruitment was best predicted by higher availability and quality of forage on summer range than winter ranges on the Kenai Peninsula, Alaska [76], indicating support for the maternal carryover hypothesis rather than the spring bottleneck or winter severity hypotheses.

We found that a later fall freeze positively impacted island-wide recruitment, supporting the maternal carryover hypothesis. The positive effects of higher quality forage in summer and fall due to increased precipitation and warmer temperatures on ungulate abundance and recruitment are well-documented [19, 72, 76]. However, little research has been conducted on how winter onset timing affects ungulate recruitment [77]. Though fall forage quality decline can be gradual, it is accelerated by sudden frost events [78]. Increased availability of fall forage over a longer time may be crucial for ungulates [79], as high-quality fall forage can increase ungulate body fat levels at the beginning of winter, therefore increasing winter survival, successful parturition, and spring calf condition [80, 81]. Additionally, warmer or longer falls allow later born calves to increase their body condition to catch up with earlier-born conspecifics, which in turn can increase their winter survival and likelihood of pregnancy as yearlings [18].

Elk recruitment declined with increasing brown bear abundance in the non-timber harvest and island-wide recruitment models, supporting the predation hypothesis. Predation by brown bears on ungulates, specifically neonates, is well documented, as are the effects of brown bear presence on elk reproductive success and calf survival [3, 20], but few studies have examined the effects of brown bear abundance on ungulate recruitment, especially in a northern system devoid of other predators. Though brown bears can be specialist predators of ungulate neonates in the spring, predation by brown bears on ungulates is likely limiting rather than regulatory, especially in higher-density ungulate populations [40]. Because brown bears often predate ungulates opportunistically [82], their role as predators varies based on the availability of ungulates and presence of alternate food [40, 83]. Brown bear predation patterns are strongly seasonal, especially in coastal populations, as brown bears switch from ungulate calves in the spring to more readily available salmon and berries during summer [84]. Though winter severity and predation may have additive effects on ungulate survival [21], we did not find that the long-term decline in elk recruitment translated to a decline in abundance, indicating that high juvenile and adult survival in mild winters may be supporting compensatory brown bear predation, even in a low-density population [7, 72, 85, 86]. The strong influence of brown bear abundance on elk recruitment indicates that ungulate populations that are small or declining may experience accelerated declines where brown bear populations are increasing [87], especially where other predator species occur, or where winter conditions are more severe [21, 86].

Area of timber harvest did not influence elk recruitment, potentially because the positive effects of forage quantity in recent timber harvest areas negated any adverse effects of reduced cover from severe weather and predation [4, 25]. Alternatively, because northern Afognak Island, where timber harvest occurs, is more heavily forested than southern Afognak Island and Raspberry Island, the lack of decline in recruitment in the timber harvest dataset and lack of a predation effect identified from the timber harvest model could indicate that mature and old-growth forests are important for elk during parturition to mitigate brown bear predation on neonates [26, 88]. During parturition, ungulates select habitat with high cover and high visibility, such as older forest stands [32, 88], which may reduce predation risk [89] and therefore decrease the effect of brown bear predation on recruitment. We recommend further research on the impacts of brown bear predation on elk calf survival, especially in areas with timber harvest.

Our conclusions are subject to consideration of some limitations. First, though the Downing population reconstruction accurately estimates population trends, it generally underestimates abundance by approximately 15% [54]. Therefore, our estimates of brown bear abundance are used only as an index of abundance. Downing population reconstruction assumes there is no variability or long-term trends in harvest rates. Though true rates of harvest relative to brown bear abundance are unknown, number of brown bears harvested varied across years and increased overall, which may have reduced precision and caused overestimation of rate of change in abundance [54]. However, the variability and increase in harvest is likely not due to changes in harvest regulations, number of hunters, or reporting rates [55]. Additionally, the estimated increase in brown bear abundance between 1993 and 2008 is consistent with documented brown bear abundance increases on Kodiak Island during that time [41].

Second, though our models fit well, we were unable to evaluate the effects of density dependence on recruitment because accurate elk abundance estimates were unavailable except for the Raspberry herd. Though in the Raspberry herd abundance model, increasing elk abundance appeared to significantly decrease growth rate and therefore abundance the following year, the effects of density dependence may be overestimated due to independent sampling error and thus weak parameter identification [66, 90, 91]. Additional analysis found no evidence of density dependence for the Raspberry herd, further limiting our conclusions regarding density dependence in this population. The effects of density dependence tend to be minimal in northern ungulate populations when predators are present [7], indicating that the parameter for density dependence may have been overestimated in our model for Raspberry herd abundance. Though Ricker and θ-logistic models may better approximate the effects of density dependence on abundance for species with slower life histories, these methods also require informative priors to provide accurate parameter estimates for density dependence [65, 92]. Furthermore, there is little difference in model fit or parameter estimates between Ricker and Gompertz models, especially when a population is below carrying capacity and the species exhibits undercompensatory population dynamics [12, 93, 94], which were both likely true for elk in our study [35, 40, 64].

Third, because over-winter and spring survival of yearlings contributes to recruitment, and yearlings are less likely to produce calves than adult females, two-month recruitment is a measure of calf survival as well as yearling and adult fecundity [44, 95]. Furthermore, our results on recruitment trends do not necessarily indicate overall abundance trends, as two-month recruitment does not measure yearling or adult survival which may drive variation in population growth among ungulate populations [96]. Yet, age ratios can be correlated with annual population growth rates, indicating that they do have value as indicators of population dynamics [3, 44]. Age class surveys are subject to observation errors due to sightability and misclassification [97] which could have led to bias in estimated relationships. However, we used composition counts conducted by experienced biologists from the same time of year and removed counts that were incomplete or where detection was compromised, reducing these errors [44]. Finally, because the number of harvest permits issued was directly determined by elk population count in the previous year, and number of elk harvested is determined by number of permits issued, elk harvest and elk abundance were intercorrelated. Therefore, though we believe that harvest likely has a significant impact on elk abundance [21, 98], we were unable to determine the effects of harvest on elk abundance or rate of elk population growth.

Forage conditions, predation, and habitat are key determinates of ungulate abundance and recruitment [6, 7]. Though drivers of ungulate population variation are species- and area-specific [96], that elk abundance increased with increasing winter mean temperatures supports the hypothesis that elk survival, and therefore abundance, is negatively affected by harsh winter conditions [3, 21]. Similarly, that recruitment increased with later first fall freeze adds to a growing body of work which indicates summer and fall forage conditions are more important than winter and spring conditions in driving ungulate vital rates [99–102], especially in subpolar maritime climates [6, 103]. However, the effects of interactions between summer-autumn nutrition, winter severity, and predation on ungulate abundance and recruitment remain unclear. Our results demonstrate a balance between the positive effects of sufficient summer-autumn nutrition and favorable winter conditions with the negative effects of predator abundance in influencing ungulate population dynamics. If ungulate abundance and recruitment are declining or low, an increase in predator abundance may cause an increase in additive predation mortality if summer-autumn nutritional conditions are poor or winters are severe [72], especially if there is limited alternative food for predators [82]. Summer and fall forage productivity is important for ungulate populations, especially in areas with increasing predator abundance, as high productivity may balance the negative effects of predation on ungulate recruitment and survival [72, 104]. Understanding the underlying mechanisms behind population trends enables us to better develop management and conservation strategies that can plan for variable responses of animals to ecosystem change including habitat alterations and predator abundance.

## Supporting information

S1 Fig Reconstructed brown bear abundance, fall mean temperature and winter mean temperature by year. Predictor data used to model elk recruitment and abundance by year for (A) reconstructed brown bear abundance with linear model and 95% confidence interval (shading; 1967–2013); (B) day of first fall freeze (1958–2020); (C) winter mean temperature (°C; 1958–2020), Afognak and Raspberry Islands, Alaska, USA.
(TIF)

S2 Fig Elk age ratios by year with linear models for timber, non-timber, and island-wide recruitment datasets. Semi-annual elk age ratio (calves per 100 adult females) data and linear models by year for (A) timber harvest (blue), (B) non-timber harvest (red), and (C) island-wide (yellow) recruitment datasets with 95% confidence intervals (shading), Afognak and Raspberry islands, Alaska, USA, 1967–2013.
(TIF)

S3 Fig Raspberry herd population counts by year with linear model. Semi-annual elk population counts and linear model by year with 95% confidence interval (shading), Raspberry Island, Alaska, USA, 1958–2020.
(TIF)

S4 Fig Elk per capita growth rate of the Raspberry herd by population count. Semi-annual elk population counts at year t (N_t_) and the natural log of the per capita population growth rate (ln(N_t_/N_t_+1)), with zero per capita growth marked with a dashed line, Raspberry Island, Alaska, USA, 1958–2020.
(TIF)

S1 Table Elk recruitment data exclusion by herd and exclusion reason. Herd indicates the name of the herd surveyed (locations shown in Fig 2), with the number of years where counts of herds were excluded from analysis because no calves were counted (zero calves), the number of calves was greater than the number of cows (calves > cows), and cows and bulls were not distinguished in the counts (adults unknown), Afognak and Raspberry islands, Alaska, USA, 1967–2017.
(DOCX)

S1 File Correlation matrix of all predictors tested on elk recruitment and abundance. Afognak and Raspberry islands, Alaska, USA, 1958–2020.
(CSV)

S2 File Annotated R code used to fit Bayesian linear (recruitment) and Gompertz (abundance) models to elk data. Models fit in JagsUI framework.
(R) Click here for additional data file.

S1 Appendix Explanation and parameterization of Bayesian linear (recruitment) and hierarchical logistic Gompertz (abundance) models. (DOCX)

S1 Dataset Brown bear harvest by year, cementum age, proportion of teeth aged, and reconstructed abundance. Shown by age class, collapsed to five age classes (St), with proportion teeth aged (At), and Downing-reconstructed abundance (Nt), Afognak and Raspberry islands, Alaska, USA, 1967–2017.
(CSV)

S2 Dataset Full dataset used to model elk recruitment and abundance, Afognak and Raspberry islands, Alaska, USA, 1958–2020. (CSV)
