# Supplementary figures and images for: Maternal carryover, winter severity, and brown bear abundance relate to elk demographics
Source: PLoS One. 2022 Sep 29;17(9):e0274359. doi: 10.1371/journal.pone.0274359 (PMC9521920; doi:10.1371/journal.pone.0274359)

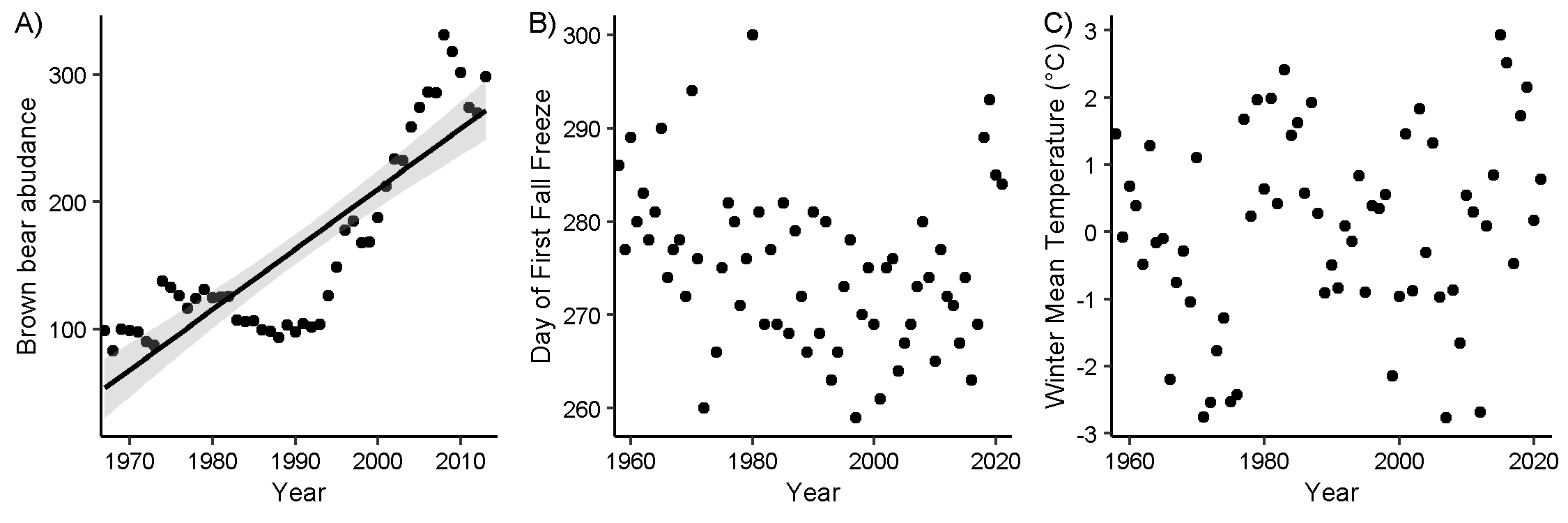

Supplement: S1 Fig — Predictor data used to model elk recruitment and abundance by year for (A) reconstructed brown bear abundance with linear model and 95% confidence interval (shading; 1967–2013); (B) day of first fall freeze (1958–2020); (C) winter mean temperature (°C; 1958–2020), Afognak and Raspberry Islands, Alaska, USA. (TIF) [file pone.0274359.s001.tif]

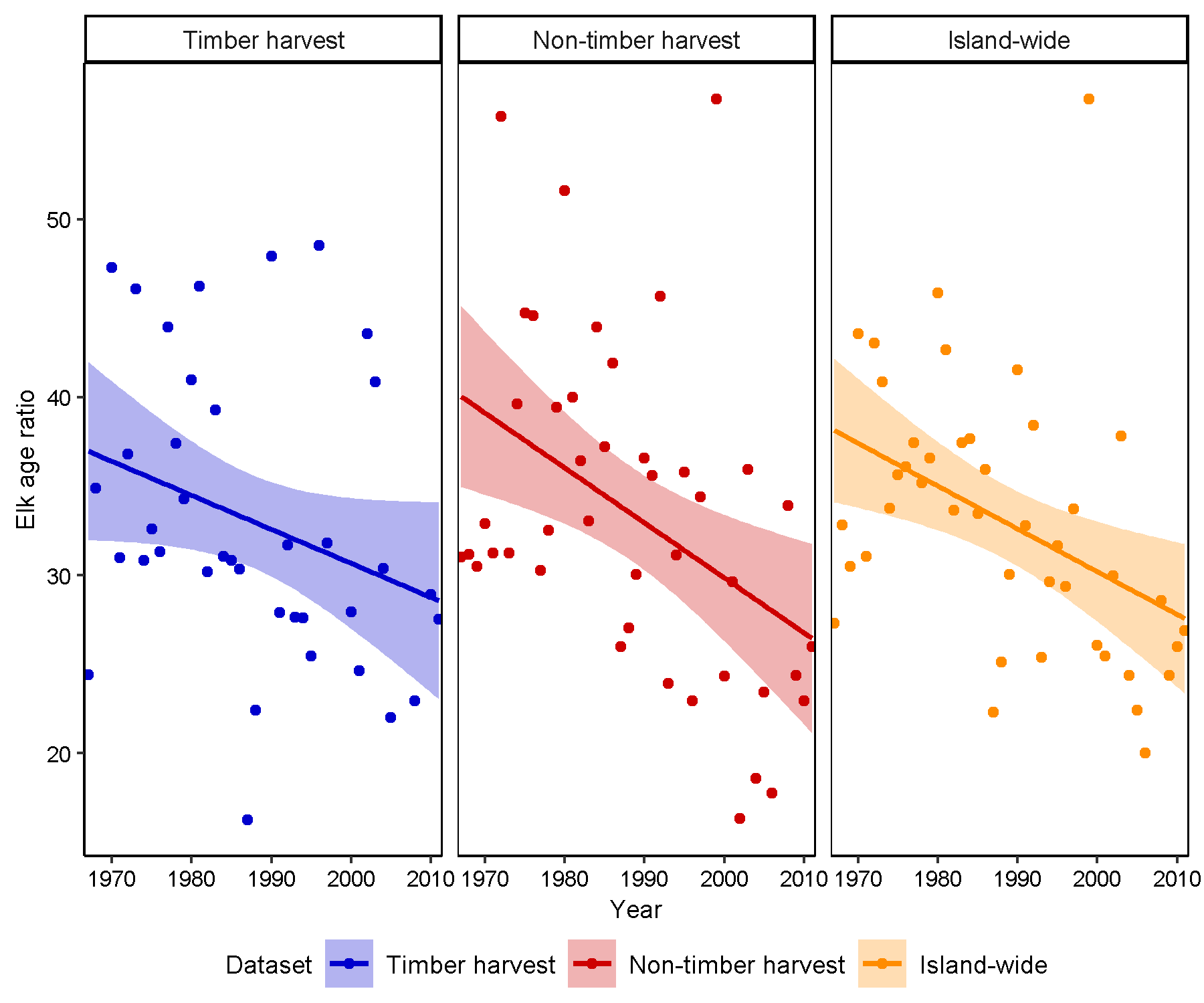

Supplement: S2 Fig — Semi-annual elk age ratio (calves per 100 adult females) data and linear models by year for (A) timber harvest (blue), (B) non-timber harvest (red), and (C) island-wide (yellow) recruitment datasets with 95% confidence intervals (shading), Afognak and Raspberry islands, Alaska, USA, 1967–2013. (TIF) [file pone.0274359.s002.tif]

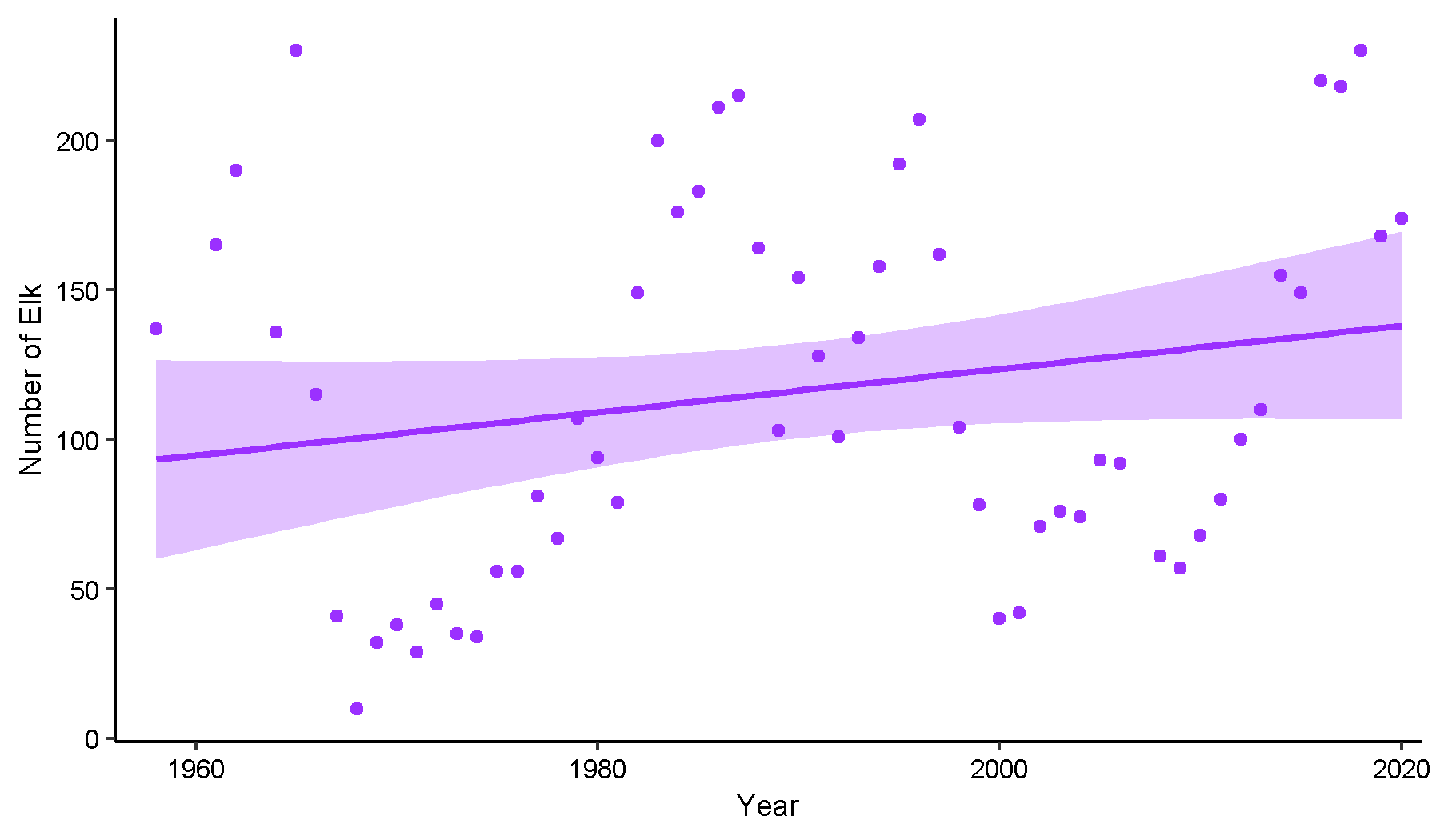

Supplement: S3 Fig — Semi-annual elk population counts and linear model by year with 95% confidence interval (shading), Raspberry Island, Alaska, USA, 1958–2020. (TIF) [file pone.0274359.s003.tif]

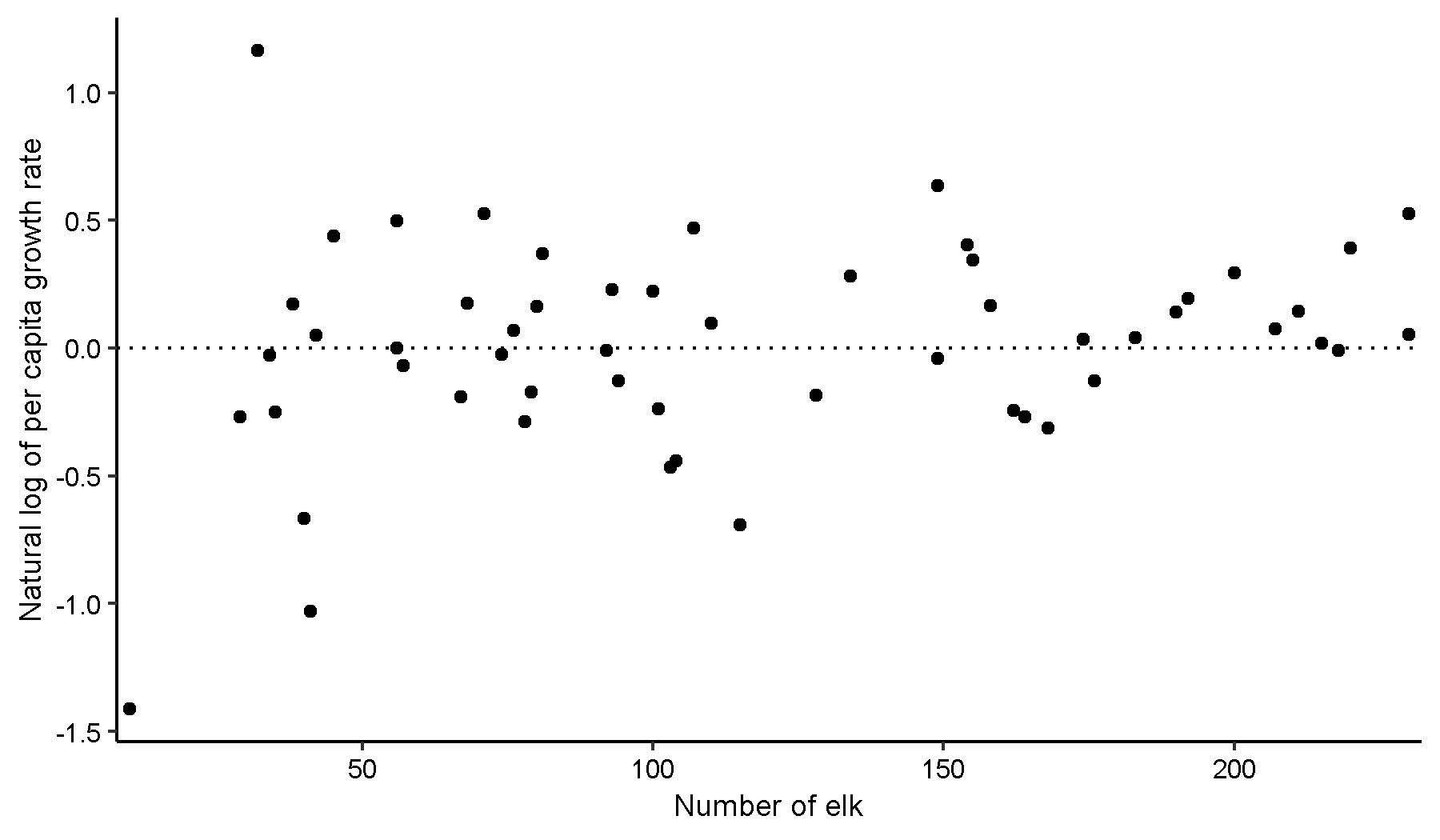

Supplement: S4 Fig — Semi-annual elk population counts at year t (Nt) and the natural log of the per capita population growth rate (ln(Nt/Nt+1)), with zero per capita growth marked with a dashed line, Raspberry Island, Alaska, USA, 1958–2020. (TIF) [file pone.0274359.s004.tif]
