# Supplementary material for: Maternal carryover, winter severity, and brown bear abundance relate to elk demographics
Source: PLoS One. 2022 Sep 29;17(9):e0274359. doi: 10.1371/journal.pone.0274359 (PMC9521920; doi:10.1371/journal.pone.0274359)
Supplement: S1 Table — Herd indicates the name of the herd surveyed (locations shown in Fig 2), with the number of years where counts of herds were excluded from analysis because no calves were counted (zero calves), the number of calves was greater than the number of cows (calves > cows), and cows and bulls were not distinguished in the counts (adults unknown), Afognak and Raspberry islands, Alaska, USA, 1967–2017. (DOCX) [file pone.0274359.s005.docx]

**S1 Table. Number of herd counts and years of data excluded from analysis for timber harvest, non-timber harvest, and all-island datasets, Afognak and Raspberry islands, Alaska, USA, 1967-2017.**

| Herd | Zero calves | Calves > Cows | Adults unknown |
| --- | --- | --- | --- |
| Raspberry | 1 |  |  |
| Malina | 1 |  | 1 |
| Duck Mountain | 4 | 1 |  |
| Waterfall | 3 |  | 1 |
| Portage | 3 |  | 2 |
| Seal Bay | 3 |  | 1 |
| Marka Lake | 2 | 2 | 1 |
| Sum | 18 | 4 | 6 |

Name of herds (herd), and the number of years where counts of herds were excluded from analysis because no calves were counted (Zero calves), the number of calves was greater than the number of cows (Calves > cows), and cows and bulls were not distinguished in the counts (Adults unknown).
