# Supplementary material for: Maternal carryover, winter severity, and brown bear abundance relate to elk demographics
Source: PLoS One. 2022 Sep 29;17(9):e0274359. doi: 10.1371/journal.pone.0274359 (PMC9521920; doi:10.1371/journal.pone.0274359)
Supplement: S1 Appendix — (DOCX) [file pone.0274359.s008.docx]

**S1 Appendix. Description, explanation, and parameterization of both Bayesian linear (recruitment datasets) and hierarchical discrete-time logistic Gompertz (abundance dataset) models.**

*Afognak and raspberry island recruitment*

We estimated the impacts of climate variables on Afognak and Raspberry island elk recruitment using a linear model formulation

*(1)* $R_{t}=\beta_{0}+\mathbf{X}_{t}\boldsymbol{\beta}_{\mathrm{predictors}}$

Where *R_t_* is predicted recruitment at time *t*, β_0_ is an estimated intercept, and **β**_predictors_ is a vector of coefficients for the selected predictors representing the winter severity, spring bottleneck, maternal carryover, predation, and timber harvest hypotheses to be estimated for **X**_t_, the corresponding model matrix predictor data. To fit the parameters of the model, we used a Bayesian framework consisting of the prediction

(2) $r_{t}\sim f(R_{t})+\sigma^{2}$

Where σ^2^ represents generic standard deviation. We used uninformative priors of σ^2^ ~ *U*(0, 100) and all β ~ *N*(0, 0.001).

*Raspberry island abundance*

We estimated the impacts of climate variables on Raspberry island elk abundance using a state-space model adapted from a model implemented on bison in Yellowstone national park [1]. The model was based on a discrete-time Gompertz model on the logarithmic scale

(3) ${log(N}_{t+1})=x_{t}=x_{t}+r+b*x_{t}$

Where *N_t_* is abundance at time *t*, *x_t_* is the natural logarithm of abundance at time *t*, *r* is the intrinsic rate of growth from low abundance (*N* = 1), and *b* is the strength of density dependence [2]. Hunting of the elk population occurred in the autumn following the survey, and therefore we implemented known harvest into Equation 1 using a post-harvest parameterization

(4) $x_{t+1}=x_{t}+h_{t}+ r+b{(x}_{t}+h_{t})+ \varepsilon_{t}$

Where *h_t_* = log|1 – *H_t_/N_t_*| is the logarithmic integration of the number of harvested individuals *H* in the season following the survey at *t.* The value if *h_t_* is zero when there are no harvested individuals and negative when *H_t_* > 0 [1,3]. We included a process error term ε ~ *N*(0*,* σ*_p_*^2^) to account for variation in population size not accounted for elsewhere in the model. To examine the effects of predictors on elk population dynamics, we modified equation (4) to include covariates for selected predictor climate variables on the growth rate:

(5) $x_{t+1}=x_{t}+e_{t}+ \beta_{0}+\beta_{1}{(x}_{t}+h_{t})+\boldsymbol{X}_{t}\boldsymbol{\beta}_{predictors}+\varepsilon_{t}$

In this new equation, β_0_ is the baseline growth rate (*r* in equation 4) β_1_ is the density dependence parameter (*b* in equation 4). **β**_predictors_ is a vector of coefficients for the selected predictors representing the winter severity, spring bottleneck, maternal carryover, predation, and timber harvest hypotheses to be estimated for **X**_t_, the corresponding model matrix predictor data. As mentioned in the main text, it must be noted that β_0_ and β_1_ are only partially identifiable without using external information to inform prior distributions [4–6], which we were hesitant to do because of the lack of previously published information on elk in this system. Finally, we modeled the log count at time *t*, *y_t_*, as the sum of *x_t_* and a normally distributed sampling error term η_t_ ~ *N*(0*,* σ*_o_*^2^):

(6) $y_{t+1}\sim g\left( x_{t+1} \right)+\eta$

The logarithm of estimated abundance at time zero was estimated with a minimally informative prior as *x_0_* ~ *U(*min(*y*), max(*y))*, and an initial value equal to *y_1_*. For observation and process error, we specified uninformative priors of σ ~ *U*(0, 10). For our parameter estimates, we specified uninformative priors of β ~ *N*(0, 0.001). Code in R programming language utilizing the JagsUI R package is provided as Supporting Information File S2.

**Literature Cited**

1. Koons DN, Colchero F, Hersey K, Gimenez O. Disentangling the effects of climate, density dependence, and harvest on an iconic large herbivore’s population dynamics. Ecol Appl. 2015;25: 956–967. doi:10.1890/14-0932.1

2. Dennis B, Ponciano JM, Lele SR, Taper ML, Staples DF. Estimating density dependence , process noise, and observation error. Ecol Monogr. 2006;76: 323–341.

3. Iijima H, Nagaike T, Honda T. Estimation of deer population dynamics using a bayesian state-space model with multiple abundance indices. J Wildl Manage. 2013;77: 1038–1047. doi:10.1002/jwmg.556

4. Clark JS, Bjørnstad ON. Population time series: process variability, observation errors, missing values, lags, and hidden states. Ecology. 2004;85: 3140–3150. doi:10.1890/03-0520

5. Delean S, Brook BW, Bradshaw CJA. Ecologically realistic estimates of maximum population growth using informed Bayesian priors. Methods Ecol Evol. 2013;4: 34–44. doi:10.1111/j.2041-210x.2012.00252.x

6. Viljugrein H, Stenseth NC, Smith GW, Steinbakk GH. Density dependence in North American ducks. Ecology. 2005;86: 245–254.
